# Synthesis and Evaluation of Non-peptidic Cysteine Protease Inhibitors of *P. falciparum* Derived from Etacrynic Acid

**DOI:** 10.3390/molecules14010019

**Published:** 2008-12-23

**Authors:** Marie-Adrienne Dude, Ulrich Kaeppler, Monika Herb, Markus Schiller, Franziska Schulz, Birgit Vedder, Saskia Heppner, Gabriele Pradel, Jiri Gut, Philip J. Rosenthal, Tanja Schirmeister, Matthias Leippe, Christoph Gelhaus

**Affiliations:** 1 Research Center for Infectious Diseases, University of Würzburg, Röntgenring 11, 97070 Würzburg, Germany; E-mail: adrienne.dude@uni-wuerzburg.de (M-A. D.), gabriele.pradel@mail.uni-wuerzburg.de (G. P.); 2 Institute of Pharmacy and Food Chemistry, Am Hubland, D-97074 Würzburg, Germany E-mail: schirmei@pharmazie.uni-wuerzburg.de (T. S.); 3 Zoological Institute, University of Kiel, Olshausenstr. 40, D-24098 Kiel, GermanyE-mail: mleippe@zoologie.uni-kiel.de (M. L.); 4 Department of Medicine, San Francisco General Hospital, University of California, San Francisco, CA 94143-0811, USA; E-mail: philip.rosenthal@ucsf.edu (P-J. R.)

**Keywords:** Malaria, Cysteine protease inhibitor, Etacrynic acid.

## Abstract

A series of etacrynic acid derivatives was synthesized and screened for their *in vitro* activity against *Plasmodium falciparum*, as well as their activity against recombinantly expressed falcipain-2 and -3. The two most active compounds of the series displayed IC_50_ values of 9.0 and 18.8 μM against *Plasmodia*.

## Introduction

Malaria is still a major cause of global human morbidity and mortality, with *Plasmodium falciparum* being the deadliest among the four *Plasmodium* species that infect humans. Annually 350–500 million clinical malaria episodes occur, causing more than 1 million deaths each year [1]. The increasing spread of *P. falciparum* due to inadequate vector control, increasing drug resistance, and the lack of effective vaccine strengthens the need for the development of novel drugs for the treatment of malaria [2, 3]. New therapeutic strategies are therefore urgently needed. Cysteine proteases of *P. falciparum* represent attractive antiplasmodial drug targets due to their essential functions for the parasite erythrocytic cycle [4, 5, 6]. Among them are falcipain-2 and falcipain-3, which are located in the acidic food vacuole of the parasite, play a pivotal role in hemoglobin hydrolysis, and may also participate in erythrocyte rupture [5, 7]. Disruption of the hemoglobin degradation pathway is lethal for the parasite. Development of compounds targeting falcipain-2 and falcipain-3 is therefore currently a focus of research. A recent study has further shown that individual cysteine protease inhibitors significantly reduce microgametogenesis in *P. falciparum*, pointing to a potential role of these inhibitors for transmission blocking strategies [8]. So far several peptidic and peptidomimetic as well as non-peptidic cysteine protease inhibitors have been synthesized and tested against malaria parasites [9,10,11,12]. In the present work a set of non-peptidic cysteine protease inhibitors derived from the loop diuretic etacrynic acid as a lead compound were evaluated for their potency to inhibit recombinantly expressed falcipain-2 and falcipain-3 [13,14,15]. Furthermore, activities against chloroquine-sensitive *P. falciparum* strain 3D7 and the chloroquine-resistant strain W2 were investigated. Several of these compounds have initially been tested against papain, the prototype cysteine protease of the CAC1 family, and against the SARS coronavirus main protease M^pro^, as described elsewhere [16, 17].

## Results and Discussion

**Scheme 1:**
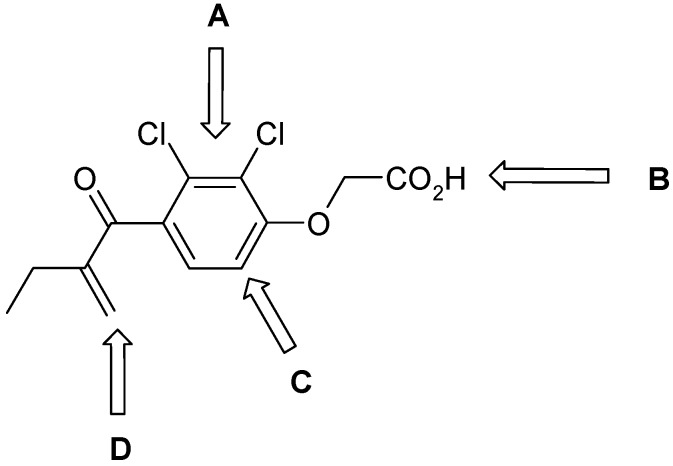
Sites of modification of the etacrynic acid lead.

In these previous studies compounds possessing an activated double bond revealed covalent, but reversible binding to the cysteine residue of the respective protease. In addition to the structural modifications implemented in previous work [16, 17], namely within the compounds **1**, **2**, **8**-**21**, we now included fluoro-substituted compounds **3**-**7**, several analogues without an activated double bond **25**-**32**, as well as derivatives with polar side chains **22**-**23**, and finally, a biotin-labeled inhibitor **24**. In summary, the structure of etacrynic acid was modified as follows (Scheme 1): **A:** substitution pattern of the aromatic ring, **B:** esters and amides, **C:** ortho-position of the double bond containing side chain (cpd. **2**), **D:** removal of the double bond.

**A:** substitution pattern of the aromatic ring; **B:** esters, amides, acids; **C:** ortho-position; **D:** removal of the double bond.

**Scheme 2:**
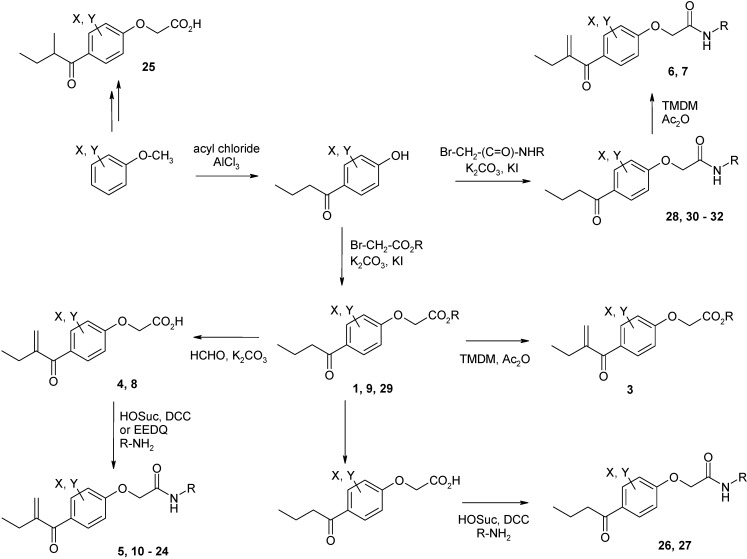
Synthetic pathways to the etacrynic acid derivatives. HOSuc, *N*-hydroxysuccinimide; DCC, dicyclohexylcarbodiimide, TMDM, *N,N,N’,N’* tetramethyldiaminomethane; EEDQ, ethyl 1,2-dihydro-2-ethoxyquinoline-1-carboxylate.

The inhibitors were synthesized according to previously described pathways [16,17] which are summarized in Scheme 2. Halogen substituted anisoles were subjected to Friedel-Crafts acylation yielding the corresponding phenolic ketones. Further alkylation of the phenolic hydroxyl functions yielded amides **28**, **30** – **32**, and esters **1**, **9** and **29**. Introduction of the double bond was performed either by Mannich reaction with TMDM (➔ **3**, **6**, **7**) or by aldol condensation with formaldehyde (➔ **4**, **8**). The latter yielded the free acids **4** and **8** due to concurrent hydrolysis of the ester function. The free acids were coupled to various amides to give the amides **5**, **10** – **24**. Amides **26** and **27** without activated double bond were also synthesized by standard amide coupling methods.

Recombinant falcipain-2 and falcipain-3 were produced as previously described [14, 18]. Inhibitory activities against recombinant falcipain-2 and falcipain-3 were evaluated in fluorometric microplate assays using the substrates Cbz-Phe-Arg-AMC and Cbz-Leu-Arg-AMC (AMC, 7-amino-4-methyl-coumarin) [19]. The cysteine protease inhibitor E-64 was used as a positive control [20]. The solvent DMSO was used as negative control. Compounds **1-6, 8-11 and 13-28** were tested *in vitro* against the CQ-sensitive 3D7 *P. falciparum* strain or the CQ-resistant W2 *P. falciparum* strain. The corresponding IC_50_ values are shown in Table 1. The *in vitro* data for the etacrynic acid derivatives are compared to those of the well known drug chloroquine and to E-64. In addition, the cytotoxicity of the inhibitor **23 **was studied on human kidney epithelium cell-line 293T, as described previously [21, 22], resulting in an IC_50_ value of >160 mM. 

**Table 1:** Inhibition of falcipain-2 / -3 (FP-2 / -3) as well as antiplasmodial activity of non-peptidic Michael-acceptors derived from etacrynic acid. 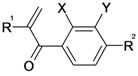

Cpd.	R^1^	X	Y	R^2^	FP-2 IC_50_, (mM)	FP-3 IC_50_, (mM)	*P. f.* 3D7/W2, IC_50 _ (µM)
**1**	H_5_C_2_	H	Cl	O-CH_2_-CO_2_Et	498±8	346±14	ni^c^
**2**	H_5_C_2_	O-CH_2_-CO_2_Et	H	Cl	110±5	381±21	ni^c^
**3**	H_5_C_2_	H	F	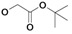	ni	nd	142±5^b^
**4**	H_5_C_2_	H	F	O-CH_2_CO_2_H	nd	nd	79.8±6^b^
**5**	H_5_C_2_	H	F	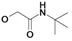	80±5	nd	205±9^b^
**6**	H_5_C_2_	H	F	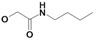	ni	nd	141±4^b^
**7**	H_5_C_2_	H	F	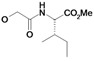	ni	nd	nd
**8**	H_5_C_2_	Cl	Cl	O-CH_2_-CO_2_H	443±17	ni	ni^c^
**9**	H_5_C_2_	Cl	Cl	O-CH_2_-CO_2_Et	60.6±4.2	163±5.6	ni^c^
**10**	H_5_C_2_	Cl	Cl	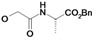	178±14	56.7±5.7	ni^c^
**11**	H_5_C_2_	Cl	Cl	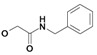	333±5	158±11	ni^c^
**12**	H_5_C_2_	Cl	Cl	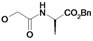	165±12	nd	nd
**13**	H5C2	Cl	Cl	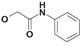	269±21	87.2±6	nic
**14**	H5C2	Cl	Cl	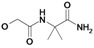	318±14	ni	29.3±3.4c
**15**	H5C2	Cl	Cl	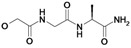	212±17	ni	nic
**16**	H5C2	Cl	Cl	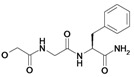	242±19	ni	nic
**17**	H5C2	Cl	Cl	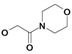	305±30	479±23	nic
**18**	H5C2	Cl	Cl	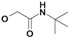	255±4	153±16	nic
**19**	H5C2	Cl	Cl	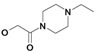	144±11	557±23	27.4±4.1c
**20**	H5C2	Cl	Cl	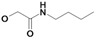	184±17	158±17	nic
**21**	H5C2	Cl	Cl	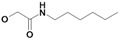	182±9	123±8	nic
**22**	H5C2	Cl	Cl	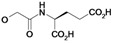	ni	ni	nic
**23**	H5C2	Cl	Cl	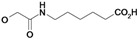	57.1±13	96.5±0.6	18.8±0.9c
**24**	H5C2	Cl	Cl	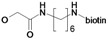	3.0±1.1	11.9±1.1	9.0±0.4c
**25**	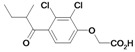				531^a^	ni	ni^c^
**26**	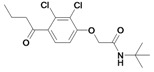				484^a^	ni	ni^c^
**27**	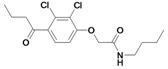				713^a^	ni	ni^c^
**28**	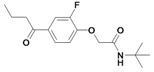				80±6	nd	66.4±2.2^c^
**29**	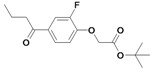				80±6	nd	nd
**30**	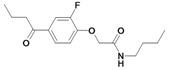				ni	nd	nd
**31**	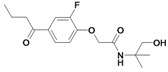				ni	nd	nd
**32**	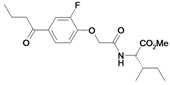				ni	nd	nd
**E-64**					0.015±0.008	0.075±0.02	5.3±1.05^c^
**CQ (W2)**					nd	nd	0.24[19]
**CQ (3D7)**					nd	nd	0.01±0.0048

^a^ Only one experiment; ni, no inhibition; nd, not determined; CQ, chloroquine; ^b ^3D7 strain; ^c^ W2 strain.

Inspection of the data in Table 1 allows the following conclusions to be drawn: in general, the etacrynic acid derivatives are weak or moderate inhibitors of falcipains and *P. falciparum*. Nevertheless, some structure-activity relationship can be found:

The ethyl ester derivative (**9**) is more potent than the etacrynic acid (**8**), both against falcipain-2 and -3. This is in accordance with the results for papain and the SARS-CoV M^pro^. The dichloro-substituted compounds (e.g. **9**, **20**) are better inhibitors than the mono-chloro- (e.g. **1**) or fluoro-substituted (**6**) compounds, with one exception, namely inhibitor **5** (compared to **18**). The α,ß-unsaturated system appears to be favourable for activity against falcipains when comparing the analogous dichloro-compounds **20** and **27** or compounds **18** and **26**. Short voluminous moieties such as the *tert*butyl moiety of compounds **5**, **28**, **29**, seem to be advantageous if combined with the fluoro-substituted aromate. In these cases, the activated double bond is apparently not essential for inhibition.

Nearly all etacrynic acid amides show better inhibition properties than the free acid (**8**). A longer acidic side-chain significantly enhances the activity (**23**, compared to **20, 21**), making **23** the most active inhibitor of falcipains and *P. falciparum* within the series. However, insertion of an additional acidic group (e.g. **22**) diminishes the inhibiting activity. The cytotoxicity/antiplasmodial ratio for the most active compound **23 **is **>**8.5, indicating selectivity against the parasite. As the data against the target enzymes and the parasites do not correlate in all cases (e.g. **14**), the question arises whether there are additional or other targets. In order to allow further affinity binding studies the biotinylated dichloro-substituted etacrynic acid amide **24** was included and synthesized according to the methods recently described [17,23] (Scheme 2). Notably, this compound emerged as the most potent inhibitor of falcipains and *P. falciparum* within the series. 

## Conclusions

In summary, this paper describes a comprehensive screening of non-peptidic Michael acceptors using etacrynic acid as lead structure. The best inhibition against recombinantly synthesized falcipain-2 and falcipain-3 revealed the compound **24**. Moreover, this etacrynic acid amide as well as compound **23** displayed modest antiplasmodial activity *in vitro* with IC_50_ values of 9 and 18.8 µM, respectively, which are in the range of the standard cysteine protease inhibitor E-64. In addition, the high IC_50_ value of >160 µM for compound **23** obtained from cytotoxicity assays using the human kidney epithelium cell-line 293T indicates selectivity against the parasite. These results provide basic information for the development of further non-peptidic irreversible cysteine protease inhibitors with etacrynic acid amides as lead compounds. In addition, the good inhibitory properties of **24** allow further affinity binding studies.

## Experimental

### General

Melting points were determined in open capillary on a melting point apparatus, model 530, from Büchi, Switzerland. NMR spectra were recorded on an AVANCE 400 MHz spectrometer from Bruker Biospin GmbH, Germany [solvent CDCl_3_ (unless otherwise noted); ^1^H-NMR, 400.13 MHz; ^13^C-NMR, 100.61 MHz]. The optical rotation values were determined on a Perkin-Elmer 241 polarimeter. ESI mass spectra were recorded on an Agilent 1100 ion trap equipped with an Agilent HPLC system. Hydrostatic column chromatography was performed with silica gel 60 (0.063-0.2 mm). All solvents were purified and dried prior to use according to standard literature procedures. Chloroquine, E64 and DMSO (dimethyl sulfoxide) were obtained from Sigma-Aldrich, Deisenhofen, Germany. Alamar Blue^®^ was purchased from Trinova Biochem, Giessen, Germany. DMEM (Dulbecco’s Modified Eagle’s Medium) high glucose was delivered from Gibco/Sigma-Aldrich, Deisenhofen, Germany. Hygromycin was obtained from Merck, Darmstadt, Germany.

### Syntheses of inhibitors

The syntheses of compounds **1**, **2**, **8-21**, and **25** are described in [17]. 

*Method A: Introduction of the double bond.* To substances 29, 30 or 32 (1 equiv.) and TMDM (*N,N,N’,N’*-tetramethyldiaminomethane, 20 equiv.), acetic anhydride (20 equiv.) was added slowly. The mixture was heated under reflux at 85 °C. The reaction is followed by ^1^H-NMR spectroscopy. After completion of the reaction the mixture was cooled to room temperature and saturated K_2_CO_3_ solution was added until gas evolution stopped. The product was extracted with Et_2_O, washed with water and brine and dried with Na_2_SO_4_. The solvent was removed *in vacuo* and the product purified by column chromatography.

*Method B: Syntheses of bromoacetamides.* Bromoacetyl bromide (1 equiv.) in absolute CH_2_Cl_2_ was cooled to -30 °C. Amine (1 equiv.) and triethylamine (1 equiv.) in absolute CH_2_Cl_2_ were added dropwise. After the addition, the reaction mixture was warmed up to room temperature and stirred for 1-2 h further. The solvent was removed *in vacuo,* the product was dissolved in acetone and filtered off. The bromoacetamides were used directly without further purification.

*Method C: Coupling of the bromoacetamide or bromoacetic acid ester with 1-(3-Fluoro-4-hydroxyphenyl)butan-1-one.* 1-(3-Fluoro-4-hydroxyphenyl)butan-1-one (1 equiv.), K_2_CO_3_ (1.5 equiv.) and KI (0.1 equiv.) were refluxed in dry acetone for 1 h. Bromoacetamide (method B) or bromoacetic acid ester (2 equiv.) were added and the mixture heated to reflux for a further 4 h. The mixture was cooled to room temperature, filtered and the solvent was removed *in vacuo*. The product was dissolved in Et_2_O, washed with brine, KOH solution (5%) and water, and dried with Na_2_SO_4_. The solvent was removed *in vacuo* and the product was purified by column chromatography.

*Tert-butyl 2-(2-fluoro-4-(2-methylenebutanoyl)phenoxy)acetate* (**3**). Method A: *Tert*-butyl-2-(4-butyryl-2-fluorophenoxy)acetate (**29**, 558 mg, 1.89 mmol), TMDM (5.14 mL, 37.7 mmol), acetic anhydride (3.56 mL, 37.7 mmol). R_f_: 0.73 (cyclohexane/ethyl acetate: 1/1); yield 180 mg (0.58 mmol, 31 %); yellow oil; LOOP-ESI-MS: calcd. for C_16_H_21_FO_4_ 308.35, found [M+H]^+^ 309.2; LC-MS: R_t_ = 38.2 min, purity 100 %; ^1^H-NMR: δ 1.09 (t, 3H, ^3^*J* = 7.5 Hz, CH_2_CH_2_C*H_3_*), 1.47 (s, 9H, C_q_(C*H_3_*)_3_), 2.45 (q, 2H, ^3^*J* = 7.4 Hz, CH_2_C*H_2_*CH_3_), 4.64 (s, 2H, OC*H_2_*C=O), 5.50 (s, 1H, C_q_=C*H_2_*), 5.74 (s, 1H, C_q_=C*H_2_*), 6.88 (dd, 1H, *J* = 8.4 Hz, C*H_arom._*), 7.54–7.59 (m, 2H, C*H_arom._*); ^13^C-NMR: δ 12.30 (C_q_CH_2_*C*H_3_), 25.51 (C_q_*C*H_2_CH_3_), 28.01 (C_q_(*C*H_3_)_3_), 66.48 (O*C*H_2_C=O), 82.95 (*C_q_*(CH_3_)_3_), 114.09 (*C*H_arom._), 117.98 (*C*H_arom._), 122.82 (C_q_=*C*H_2_), 126.52 (*C*H_arom._), 131.64 (*C_q_*C=O), 149.39 (*C_q_*=CH_2_), 150.58 (*C_q_*F or *C_q_*OCH_2_C=O), 153.06 (*C_q_*F or *C_q_*OCH_2_C=O), 166.96 (OCH_2_*C*=O), 196.12 (C_q_*C*=O).

*2-(2-fluoro-4-(2-methylenebutanoyl)phenoxy)acetic acid* (4): *1-(3-fluoro-4-hydroxyphenyl)butan-1-one.* 2-Fluoroanisole (4.00 g, 31.7 mmol) and butyric acid chloride (5.07 g, 47.6 mmol) were dissolved under a N_2_ atmosphere in absolute CH_2_Cl_2 _(50 mL) and the mixture was cooled to 0-10 °C. AlCl_3_ (6.35 g, 47.6 mmol) was added within 30 minutes, and the mixture was stirred for 2-3 h. An additional amount of AlCl_3_ (6.35 g, 47.6 mmol) was added, and the mixture was heated under reflux for 2 h. The mixture was poured on ice and acidified with concentrated HCl to pH 1. Tartaric acid was added for complexation of aluminum until the solution was clear. The solution was extracted with Et_2_O and the organic layer was washed with KOH solution (10%) and brine. The organic layer was dried with Na_2_SO_4_ and solvent was removed *in vacuo*. Yield 4.97 g (27.3 mmol, 86 %); white solid; mp. 91 °C (water); ^1^H-NMR: δ 0.98 (t, 3H, ^3^*J* = 7.3 Hz, CH_2_CH_2_C*H_3_*), 1.74 (sext, 2H, ^3^*J* = 7.3 Hz, CH_2_C*H_2_*CH_3_), 2.87 (t, 2H, ^3^*J* = 7.3 Hz, C*H_2_*CH_2_CH_3_), 6.04 (bs, 1H, OH), 7.04 (dd, 1H, *J* = 8.5 Hz, C*H_arom._*), 7.67–7.73 (m, 2H, C*H_arom._*).

*Ethyl-2-(4-butyryl-2-fluorophenoxy)acetate.* 1-(3-Fluoro-4-hydroxyphenyl)butan-1-one (2.00 g, 11.0 mmol), bromoacetic acid ethyl ester (3.67 g, 22.0 mmol), K_2_CO_3_ (2.28 g, 16.5 mmol) and KI (183 mg, 1.10 mmol) were refluxed in dry acetone for 5-6 h. The reaction mixture was cooled to room temperature and filtered off. The solvent was removed *in vacuo* and the product was extracted with Et_2_O, washed with NaOH solution (10%), water and brine, dried with Na_2_SO_4_, and the solvent was removed *in vacuo*. Yield 2.30 g (8.60 mmol, 78%); white solid; mp. 79-80 °C (Et_2_O). ^1^H-NMR (DMSO-d_6_): δ 0.90 (t, 3H, ^3^*J* = 7.5 Hz, CH_2_CH_2_C*H_3_*), 1.20 (t, 3H, ^3^*J* = 7.2 Hz, OCH_2_C*H_3_*), 1.60 (sext, 2H, ^3^*J* = 7.3 Hz, CH_2_C*H_2_*CH_3_), 2.93 (t, 2H, ^3^*J* = 7.1 Hz, C*H_2_*CH_2_CH_3_), 4.17 (q, 2H, ^3^*J* = 7.1 Hz, OC*H_2_*CH_3_), 4.99 (s, 2H, OC*H_2_*C=O), 7.20 (dd, 1H, *J* = 8.5, C*H_arom._*), 7.74–7.78 (m, 2H, C*H_arom._*).

*2-(2-fluoro-4-(2-methylenebutanoyl)phenoxy)acetic acid* (**4**). Ethyl-2-(4-butyryl-2-fluorophenoxy)-acetate (2.11 g, 7.87 mmol), formaldehyde solution (40%, 1.08 mL, 15.7 mmol) and K_2_CO_3_ (2.17 g, 15.7 mmol, dissolved in water) were refluxed in ethanol for 24 h. The reaction mixture was cooled to room temperature, acidified with concentrated HCl to pH 1 and the product was extracted with Et_2_O. The solvent was removed *in vacuo*. Yield 556 mg (2.20 mmol, 28%); white solid; mp 99-101 °C (Et_2_O); LOOP-ESI-MS: calcd. for C_13_H_13_FO_4_ 252.24, found [M+H]^+^ 253.1; LC-MS: R_t_ = 23.8 min, purity 100 %;^ 1^H-NMR: δ 1.10 (t, 3H, ^3^*J* = 7.5 Hz, C_q_CH_2_C*H_3_*). 2.46 (q, 2H, ^3^*J* = 7.4 Hz, C_q_C*H_2_*CH_3_), 4.81 (s, 2H, OC*H_2_*C=O), 5.52 (s, 1H, C_q_=C*H_2_*), 5.78 (s, 1H, C_q_=C*H_2_*), 6.95 (dd, 1H, *J* = 8.4 Hz, C*H_arom._*), 7.56–7.60 (m, 2H, C*H_arom._*); ^13^C-NMR: δ 12.30 (C_q_CH_2_*C*H_3_), 25.44 (C_q_*C*H_2_CH_3_), 65.65 (O*C*H_2_C=O), 114.50 (*C*H_arom*.*_), 118.19 (*C*H_arom*.*_), 123.35 (C_q_=*C*H_2_), 126.59 (*C*H_arom*.*_), 132.35 (*C_q_*C=O), 148.87 (*C_q_*=CH_2_), 149.33 (*C_q_*F or *C_q_*OCH_2_C=O), 153.11 (*C_q_*F or *C_q_*OCH_2_C=O), 172.29 (OCH_2_*C*=O), 196.16 (C_q_*C*=O).

*N-tert-butyl-2-(2-fluoro-4-(2-methylenebutanoyl)phenoxy)acetamide* (**5**). *2*-(2-Fluoro-4-(2-methylene-butanoyl)phenoxy)acetic acid (**4**, 767 mg, 3.00 mmol) and *N*-hydroxysuccinimide (336 mg, 3.00 mmol) were dissolved under a N_2_ atmosphere in absolute CH_2_Cl_2 _(10 mL). Dicyclohexylcarbodiimide (DCC) (619 mg, 3.00 mmol) was added in small portions and the mixture was stirred overnight. The mixture was filtered into a flask with *tert*-butylamine (219 mg, 3.00 mmol) in CH_2_Cl_2 _(10 mL), stirred for 3 h at room temperature and filtered. The reaction mixture was washed with brine and KOH solution (5%), dried with Na_2_SO_4_ and the solvent was removed *in vacuo*. The product was purified by column chromatography. R_f_: 0.43 (cyclohexane/ethyl acetate: 2/1); yield 314 mg (1.02 mmol, 34 %); white solid; mp 85 °C (cyclohexane/ethyl acetate); LOOP-ESI-MS: calcd. for C_17_H_22_FNO_3_ 307.37, found [M+H]^+^ 308.4; LC-MS: R_t_ = 18.0 min, purity 100 %; ^1^H-NMR: δ 1.10 (t, 3H, ^3^*J* = 7.5 Hz, C_q_CH_2_C*H_3_*), 1.40 (s, 9H, C_q_(C*H_3_*)_3_), 2.46 (q, 2H, ^3^*J* = 7.4 Hz, C_q_C*H_2_*CH_3_), 4.46 (s, 2H, OC*H_2_*C=O), 5.51 (s, 1H, C_q_=C*H_2_*), 5.78 (s, 1H, C_q_=C*H_2_*), 6.43 (bs, 1H, N*H*), 6.96 (dd, 1H, *J* = 8.2 Hz, C*H_arom._*), 7.56–7.62 (m, 2H, C*H_arom._*); ^13^C-NMR: δ 12.30 (C_q_CH_2_*C*H_3_), 25.45 (C_q_*C*H_2_CH_3_), 28.71 (C_q_(*C*H_3_)_3_), 51.46 (*C_q_*(CH_3_)_3_), 68.50 (O*C*H_2_C=O), 114.05 (*C*H_arom*.*_), 117.73 (*C*H_arom*.*_), 123.23 (C_q_=*C*H_2_), 126.92 (*C*H_arom*.*_), 132.23 (*C_q_*C=O), 146.73 *C_q_*=CH_2_), 148.87 (*C_q_*F or *C_q_*OCH_2_C=O), 149.35 (*C_q_*F or *C_q_*OCH_2_C=O), 165.95 (OCH_2_*C*=O), 196.01 (C_q_*C*=O). 

*N-butyl-2-(2-fluoro-4-(2-methylenebutanoyl)phenoxy)acetamide* (**6**). Method A: *N*-butyl-2-(4-butyryl-2-fluorophenoxy)acetamide (**30**) (105 mg, 0.36 mmol), TMDM (0.97 mL, 7.11 mmol), acetic anhydride (0.67 mL, 7.11 mmol). R_f_: 0.59 (cyclohexane/ethyl acetate: 1/2); yield 55.5 mg (0.18 mmol, 51 %); white solid; mp 73 °C (cyclohexane/ethyl acetate); LOOP-ESI-MS: calcd. for C_17_H_22_FNO_3_ 307.37; found [M+H]^+^ 308.4; LC-MS: R_t_ = 15.8 min, purity 100 %; ^1^H-NMR: δ 0.93 (t, 3H, ^3^J = 7.4 Hz, NHCH_2_CH_2_CH_2_CH_3_), 1.11 (t, 3H, ^3^*J* = 7.4 Hz, C_q_CH_2_CH_3_), 1.36 (sext, 2H, ^3^*J* = 7.4 Hz, NHCH_2_CH_2_CH_2_CH_3_), 1.53 (sext, ^3^*J* = 7.3 Hz, NHCH_2_CH_2_CH_2_CH_3_), 2.46 (q, 2H, ^3^*J* = 7.5 Hz, C_q_CH_2_CH_3_), 3.37 (q, 2H, ^3^*J* = 6.7 Hz, NHCH_2_CH_2_CH_2_CH_3_), 4.57 (s, 2H, OCH_2_C=O), 5.51 (s, C_q_=CH_2_), 5.78 (s, C_q_=CH_2_), 6.60 (s, 1H, NH), 6.96 (dd, 1H, *J* = 8.1 Hz, CH_arom._), 7.57–7.62 (m, 2H, CH_arom._); ^13^C-NMR: δ 12.31 (C_q_CH_2_CH_3_), 13.69 (NHCH_2_CH_2_CH_2_CH_3_), 20.00 (NHCH_2_CH_2_CH_2_CH_3_), 25.45 (C_q_CH_2_CH_3_), 31.53 (NHCH_2_CH_2_CH_2_CH_3_), 38.90 (NHCH_2_CH_2_CH_2_CH_3_), 68.21 (OCH_2_C=O), 113.90 (CH_arom._), 117.77 (CH_arom._), 123.22 (C_q_=CH_2_), 126.92 (CH_arom._), 149 (C_q_F or C_q_OCH_2_C=O), 166.77 (OCH_2_C=O).

*Methyl-2-(2-(2-fluoro-4-(2-methylenebutanoyl)phenoxy)acetamido)-3-methylpentanoate* (**7**). Method A: Methyl-2-(2-(4-butyryl-2-fluorophenoxy)acetamido)-3-methylpentanoate (**32**, 58.0 mg, 0.16 mmol), TMDM (0.43 mL, 3.16 mmol), acetic anhydride (0.30 mL, 3.16 mmol). R_f_: 0.75 (cyclohexane/ethyl acetate: 1/1); yield 41.5 mg (0.11 mmol, 68 %); colorless oil; LOOP-ESI-MS: calcd. for C_20_H_26_FNO_5_ 379.43; found [M+H]^+^ 380.4; LC-MS: R_t_ = 18.9 min, purity 100 %; [α]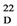: 61.1 (CHCl_3_, c = 0.1). ^1^H-NMR: δ 0.90–0.94 (m, 6H, Ile-CH_2_C*H_3_* and Ile-CHC*H_3_*), 1.11 (t, 3H, ^3^*J* = 7.5 Hz, C_q_CH_2_C*H_3_*), 1.14–1.22 (m, 2H, Ile-C*H_2_*CH_3_), 1.40–1.48 (m, 1H, Ile-C=O), 1.93–1.99 (m, 1H, Ile-C*H*CH_3_), 2.47 (t, 2H, ^3^*J* = 7.3 Hz, C_q_C*H_2_*CH_3_), 3.74 (s, 3H, OC*H_3_*), 4.62 (s, 2H, OC*H_2_*C=O), 5.51 (s, C_q_=C*H_2_*), 5.78 (s, C_q_=C*H_2_*), 6.98 (dd, 1H, ^3^*J* = 8.2 Hz, C*H_arom._*), 7.08 (d, 1H, *J* = 8.5 Hz, N*H*), 7.57–7.63 (m, 2H, C*H_arom._*); ^13^C-NMR: δ 11.51 (Ile-CH_2_*C*H_3_ or Ile-CH*C*H_3_), 12.31 (C_q_CH_2_*C*H_3_), 15.50 (Ile-CH_2_*C*H_3_ or Ile-CH*C*H_3_), 25.08 (C_q_*C*H_2_CH_3_ or Ile-*C*HC=O), 25.43 (C_q_*C*H_2_CH_3 _or Ile-N*C*HC=O), 37.79 (Ile-*C*HCH_3_), 52.23 (O*C*H_3_), 68.32 (O*C*H_2_C=O), 114.25 (*C*H_arom._), 117.86 (*C*H_arom._), 123.26 (C_q_=*C*H_2_), 126.81 (*C*H_arom._), 132.46 (*C_q_*C=O), 149.37 (*C_q_*OCH_2_C=O), 150.66 (*C_q_*=CH_2_), 153.14 (*C_q_*F), 166.83 (OCH_2_*C=*O), 171.68 (*C_q_*OCH_3_), 195.98 (C_q_*C*=O).

*2-{2-[2,3-Dichloro-4-(2-methylene-butyryl)-phenoxy]acetylamino}pentanedioic acid* (**22**). Compound **22** was synthesized according to [17] starting from etacrynic acid (240 mg, 0.79 mmol), *N*-hydroxy-succinimide (91 mg, 0.79 mmol), DCC (163 mg, 0.79 mmol) in THF. The solvent was removed and a solution of glutamic acid (116 mg, 0.79 mmol) and KOH (133 mg, 2.37 mmol) in water was added. After stirring 7 days at room temperature the mixture was acidified with conc. HCl and extracted with dichloro­methane. The crude product, which still contained etacrynic acid, was suspended in hot benzene and filtered. The solid residue yielded the pure amide as a white powder. Yield: 55 mg (0.127 mmol, 16 %). ^1^H-NMR (*d_4_*-MeOH): δ = 1.14 (t, 3H, *^3^J* = 8.0 Hz, CH_2_C*H_3_*), 2.05 (m, 1H), 2.27 (m, 1H), 2.38 – 2.47 (m, 4H, 2 CH_2_), 4.57 (m, 1H, NHC*H*), 4.75 (s, 2H, OCH_2_CO), 5.60 (s, 1H, C=CH), 6.02 (s, 1H, C=CH), 7.10 (d, 1H, *^3^J* = 8.0 Hz, Ar-H), 7.24 (d, 1H, *^3^J* = 8.0 Hz, Ar-H); LC-MS (negative mode): *m/z*: 431.2 (100) [M-H]^-^, R_t_ = 2.3 min.

*6-{2-[2,3-Dichloro-4-(2-methylene-butyryl)-phenoxy]acetylamino}-hexanoic acid* (**23**). Compound **23** was synthesized according to [17] starting from etacrynic acid (201 mg, 0.66 mmol), *N*-hydroxy-succinimide (76 mg, 0.66 mmol), DCC (137 mg, 0.66 mmol) in dichloromethane (20 mL). The solvent was removed and the residue taken up in THF. This solution was added to a solution of 6-amino-hexanoic acid (130 mg, 0.99 mmol) and KOH (111 mg, 1.98 mmol) in water (10 mL). After 3 days stirring at room temperature the mixture was acidified with conc. HCl and extracted with dichloro­methane. After removal of the organic solvent the crude product yielded a yellow oil which was purified by column chromatography (MeOH/CHCl_3_ 10:2). Product: colourless oil, which solidified to a white solid. Yield: 93 mg (0.22 mmol, 34 %); ^1^H-NMR (*d_4_*-MeOH): δ = 1.14 (t, 3H, *^3^J* = 8.0 Hz, CH_2_C*H_3_*), 1.33 – 1.40 (m, 2H, CH_2_), 1.53 – 1.66 (m, 4H, 2 CH_2_), 2.28 (t, 2H, *^3^J* = 8.0 Hz, CH_2_), 2.44 (q, 2H, *^3^J* = 8.0 Hz, C*H_2_*CH_3_), 3.30 (m, 2H, NHC*H_2_*), 4.68 (s, 2H, OCH_2_CO), 5.59 (s, 1H, C=CH), 6.03 (s, 1H, C=CH), 7.07 (d, 1H, *^3^J* = 8.0 Hz, Ar-H), 7.24 (d, 1H, *^3^J* = 8.0 Hz, Ar-H); ^13^C-NMR (*d_4_*-MeOH): δ = 13.0 (CH_3_), 24.5 (CH_2_), 25.7 (CH_2_), 27.4 (CH_2_), 30.1 (CH_2_), 34.9 (CH_2_), 40.0 (CH_2_), 69.5 (O*C*H_2_CO), 112.9 (Ar-CH), 124.0 (Ar-qC), 128.5 (Ar-CH), 129.9 (C=*C*H_2_), 131.8 (Ar-qC), 135.0 (Ar-qC), 151.7 (qC, *C*=CH_2_), 156.9 (Ar-qC), 169.7 (qC, NHCONH), 177.6 (qC, COOH), 197.3 (qC, C=O); LC-MS: *m/z*: 418.0 (15), R_t_ = 0.5 min.

### 5-(2-Oxo-hexahydro-thieno[3,4-d]imidazol-4-yl)pentanoic acid (6-{2-[2,3-dichloro-4-(2-methylene-butyryl)-phenoxy]acetylamino}-hexyl)-amide (**24**)

*(A) Coupling of Boc-diaminohexane and D-biotin:* Boc-diaminohexane (443 mg, 2.05 mmol) and D-biotin (500 mg, 2.05 mmol) were suspended in absolute DMF (45 mL) and cooled to 0 °C. DPPA (620 mg, 2.25 mmol) and triethylamine (229 mg, 2.25 mmol) were added and the mixture was stirred at 4 °C for 8 days. Dichloromethane (25 mL) was added and the organic phase was washed twice with citric acid (10%). Upon addition of water to the organic phase the amide precipitated as a white solid which was filtered off.

*(B) Removal of the Boc-protecting group:* The solid obtained in step (A) was suspended in dichloromethane (9 mL) and treated with TFA (3 mL) at 0 °C. Excess TFA was repeatedly removed *in vacuo*. For better evaporation dichloromethane was added to the residue. Quantitative removal of the protecting group was verified by ^1^H-NMR. 

*(C) Amide coupling to etacrynic acid:* Biotin-(6-amino)-hexylamide (TFA-salt) (175 mg, 0.383 mmol), triethylamine (39 mg, 0.383 mmol) and etacrynic acid (232 mg, 0.767 mmol) were dissolved in DMF (2 mL). EEDQ (190 mg, 0.767 mmol) was added and the mixture was stirred at room temperature for 14 days. Ethyl acetate (50 mL) was added. Treatment of the organic phase with Na_2_CO_3_-solution (2%, 25 mL) yielded the desired product as a white precipitate which was filtered off. Overall yield: 62 mg (0.0988 mmol) as a white solid; ^1^H-NMR (DMSO-*d_6_*): δ = 1.07 (t, 3H, *^3^J* = 7.4 Hz, CH_2_C*H_3_*), 1.23 – 1.57 (m, 14H, CH_2_), 2.03 (t, 2H,*^ 3^J* = 7.2 Hz, CH_2_), 2.36 (q, 2H, *^3^J* = 7.4 Hz, C*H_2_*CH_3_), 2.56 (d, 1H, *^3^J* = 12.3 Hz, SCH), 2.80 (dd, 1H, *^3^J* = 12.4 Hz, *^2^J* = 4.8 Hz, SCH), 2.97 – 3.12 (m, 5H, 2 NHC*H*_2_, SCH), 4.11 (m, 1H, NHC*H*), 4.29 (m, 1H, NHC*H*), 4.70 (s, 2H, OCH_2_CO), 5.55 (s, 1H, C=CH), 6.06 (s, 1H, C=CH), 6.33 (s, 1H, biotin-NH), 6.39 (s, 1H, biotin-NH), 7.07 (d, 1H, *^3^J* = 8.6 Hz, Ar-H), 7.32 (d, 1H, *^3^J* = 8.6 Hz, Ar-H), 7.70 (br s, 1H, CONH), 8.01 (br s, 1H, CONH); ^13^C-NMR (DMSO-*d_6_*): δ = 12.3 (CH_3_), 22.9 (*C*H_2_CH_3_), 25.3 (CH_2_), 26.0 (CH_2_), 26.0 (CH_2_), 28.0 (CH_2_), 28.2 (CH_2_), 28.9 (CH_2_), 29.1 (CH_2_), 35.2 (CH_2_), 38.2 (CH_2_), 38.3 (CH_2_), 39.8 (SCH_2_), 55.4 (SCH), 59.2 (NHCH), 61.0 (NHCH), 67.9 (O*C*H_2_CO), 111.9 (Ar-CH), 121.2 (Ar-qC), 127.4 (Ar-CH), 129.3 (C=*C*H_2_), 129.9 (Ar-qC), 132.4 (Ar-qC), 149.3 (qC, *C*=CH_2_), 155.5 (Ar-qC), 162.6 (qC, NHCONH), 166.3 (qC, CONH), 171.7 (qC, CONH), 195.1 (qC, C=O); LC-MS: *m/z* 628.0 (100) [M+H]^+^, R_t_ = 16.3 min.

*N-tert-butyl-2-(4-butyryl-2,3-dichloro-phenoxy)-acetamide* (**26**). Compound **26** was synthesized according to [17] starting from 2,3-dichloro-4-butyrylphenoxyacetic acid (500 mg, 1.72 mmol), *N*-hydroxysuccinimide (198 mg, 1.72 mmol), DCC (354 mg, 1.72 mmol) and *t*-butylamine (251 mg, 3.43 mmol) in dichloromethane (15 mL). Reaction time: 21 d stirring at room temperature. The crude product was purified by column chromatography (SiO_2_, Cy/EtOAc 1:1). Yield: 374 mg (1.08 mmol, 63 %) as white solid; ^1^H-NMR: δ = 0.97 (t, 3H, *^3^J* = 7.4 Hz, CH_2_C*H_3_*), 1.41 (s, 9H, (CH_3_)_3_), 1.72 (sext, 2H, *^3^J* = 7.3 Hz, C*H_2_*CH_3_), 2.88 (t, 2H, *^3^J* = 7.2 Hz, COC*H_2_*CH_2_CH_3_), 4.44 (s, 2H, OCH_2_), 6.62 (br s, 1H, NH), 6.84 (d, 1H, *^3^J* = 8.6 Hz, Ar-H), 7.38 (d, 1H, *^3^J* = 8.6 Hz, Ar-H). ^13^C-NMR: δ = 13.7 (CH_2_*C*H_3_), 17.8 (*C*H_2_CH_3_), 28.7 (C(*C*H_3_)_3_), 44.7 (CO*C*H_2_), 51.5 (*C*(CH_3_)_3_), 68.4 (OCH_2_), 111.1 (Ar-CH), 123.2 (Ar-qC), 127.6 (Ar-CH), 131.4 (Ar-qC), 134.9 (Ar-qC), 155.2 (Ar-qC), 165.5 (qC, CONH), 201.8 (C=O).

*N-butyl-2-(4-butyryl-2,3-dichloro-phenoxy)-acetamide* (**27**). Compound **27** was synthesized according to [17] starting from 2,3-dichloro-4-butyrylphenoxyacetic acid (500 mg, 1.72 mmol), *N*-hydroxy-succinimide (198 mg, 1.72 mmol), DCC (354 mg, 1.72 mmol) and *n*-butylamine (251 mg, 3.43 mmol) in dichloromethane (15 mL). Reaction time: 21 d stirring at room temperature. The crude product was purified by column chromatography (SiO_2_, Cy/EtOAc 1:1). Yield: 270 mg (0.880 mmol, 45 %) as a white solid; ^1^H-NMR: δ = 0.92 – 0.98 (m, 6H, NH(CH_2_)_3_C*H_3_*, COCH_2_CH_2_C*H_3_*), 1.38 (sext, 2H, *^3^J* = 7.4 Hz, NHCH_2_CH_2_C*H_2_*CH_3_), 1.55 (quint, 2H, *^3^J* = 7.3 Hz, NHCH_2_C*H_2_*CH_2_CH_3_), 1.72 (sextett, 2H, *^3^J* = 7.3 Hz, COCH_2_C*H_2_*CH_3_), 2.88 (t, 2H, *^3^J* = 7.3 Hz, COC*H_2_*CH_2_CH_3_), 3.37 (q, 2H, *^3^J* = 6.7 Hz, NHC*H_2_*), 4.55 (s, 2H, OCH_2_), 6.72 (br s, 1H, NH), 6.85 (d, 1H, *^3^J* = 8.8 Hz, Ar-H), 7.38 (d, 1H, *^3^J* = 8.6 Hz, Ar-H); ^13^C-NMR: δ = 13.7 (*n*-butyl-*C*H_3_, CH_2_*C*H_3_), 17.8 (*C*H_2_CH_3_), 20.0 (NHCH_2_CH_2_*C*H_2_CH_3_), 31.5 (NHCH_2_*C*H_2_CH_2_CH_3_), 38.9 (NHCH_2_), 44.8 (CO*C*H_2_CH_2_CH_3_), 68.2 (OCH_2_), 111.0 (Ar-CH), 123.3 (Ar-qC), 127.6 (Ar-CH), 131.4 (Ar-qC), 135.0 (Ar-qC), 155.1 (Ar-qC), 166.4 (qC, CONH), 201.8 (C=O).

*N-tert-butyl-2-(4-butyryl-2-fluorophenoxy)acetamide* (**28**). Method B: Bromoacetyl bromide (2.94 g, 14.6 mmol), *tert*-butylamine (1.07 mg, 14.6 mmol), triethylamine (2.05 mL, 14.6 mmol); Method C: 1-(3-fluoro-4-hydroxyphenyl)butan-1-one (856 mg, 4.70 mmol), K_2_CO_3_ (974 mg, 7.05 mmol), KI (78.0 mg, 0.47 mmol), 2-bromo-*N*-*tert*-butylacetamide (see method B). R_f_: 0.31 (cyclohexane/ethyl acetate: 2/1); yield 1.00 g (3.38 mmol, 72%); white solid; mp 80 °C (cyclohexane/ethyl acetate). ^1^H-NMR: δ 0.99 (t, 3H, ^3^*J* = 7.5 Hz, CH_2_CH_2_C*H_3_*), 1.37 (s, 9H, C_q_(C*H_3_*)_3_), 1.75 (sext, 2H, ^3^*J* = 7.3 Hz, CH_2_C*H_2_*CH_3_), 2.88 (t, 2H, ^3^*J* = 7.2 Hz, C*H_2_*CH_2_CH_3_), 4.46 (s, 2H, OC*H_2_*C=O), 6.41 (bs, 1H, N*H*), 6.97 (dd, 1H, *J* = 8.4 Hz, C*H_arom._*), 7.72–7.76 (m, 2H, C*H_arom._*).

*Tert-butyl-2-(4-butyryl-2-fluorophenoxy)acetate* (**29**). Method C: 1-(3-Fluoro-4-hydroxyphenyl)butan-1-one (1.00 g, 5.49 mmol), K_2_CO_3_ (1.19 g, 8.64 mmol), KI (91.0 mg, 0.55 mmol), bromoacetic acid *tert*-butylester (1.50 mL, 10.2 mmol); R_f_: 0.69 (cyclohexane/ethyl acetate: 1/1); yield 1.46 g (4.93 mmol, 90 %); white solid; mp 62 °C (cyclohexane/ethyl acetate); ^1^H-NMR: δ 0.98 (t, 3H, ^3^*J* = 7.3 Hz, CH_2_CH_2_C*H_3_*), 1.47 (s, 9H, C_q_(C*H_3_*)_3_), 1.74 (sext, 2H, ^3^*J* = 7.3 Hz, CH_2_C*H_2_*CH_3_), 2.86 (t, 2H, ^3^*J* = 7.2 Hz, C*H_2_*CH_2_CH_3_), 4.64 (s, 2H, OC*H_2_*C=O), 6.89 (dd, 1H, *J* = 8.5 Hz, C*H_arom._*), 7.68–7.72 (m, 2H, C*H_arom._*). 

*N-butyl-2-(4-butyryl-2-fluorophenoxy)acetamide* (**30**). Method B: Bromoacetyl bromide (2.08 g, 10.3 mmol), *n*-butylamine (753 mg, 10.3 mmol), triethylamine (1.45 mL, 10.3 mmol). Method C: 1-(3-Fluoro-4-hydroxyphenyl)butan-1-one (750 mg, 4.12 mmol), K_2_CO_3_ (853 mg, 6.17 mmol), KI (68.4 mg, 0.41 mmol), 2-bromo-*N*-n-butylacetamide (see method B). R_f_: 0.32 (cyclohexane/ethyl acetate: 1/2); yield 1.00 g (3.39 mmol, 82 %); white solid; mp 75 °C (cyclohexane/ethyl acetate); ^1^H-NMR: δ 0.93 (m, 3H, NHCH_2_CH_2_CH_2_C*H_3_*), 0.99 (t, 3H, ^3^*J* = 7.4 Hz, CH_2_CH_2_C*H_3_*), 1.36 (sext, 2H, ^3^*J* = 7.3 Hz, NHCH_2_CH_2_C*H_2_*CH_3_), 1.53 (sext, ^3^*J* = 7.3 Hz, NHCH_2_C*H_2_*CH_2_CH_3_), 1.75 (sext, 2H, ^3^*J* = 7.3 Hz, CH_2_C*H_2_*CH_3_), 2.88 (t, 2H, ^3^*J* = 7.3 Hz, C*H_2_*CH_2_CH_3_), 3.36 (q, 2H, ^3^*J* = 6.8 Hz, NHC*H_2_*CH_2_CH_2_CH_3_), 4.57 (s, 2H, OC*H_2_*C=O), 6.59 (s, 1H, N*H*), 6.97 (dd, 1H, *J* = 8.3 Hz, C*H_arom._*), 7.72–7.76 (m, 2H, C*H_arom._*); ^13^C-NMR: δ 13.68 und 13.82 (CH_2_CH_2_C*H_3_* und NHCH_2_CH_2_CH_2_C*H_3_*), 17.76 (CH_2_C*H_2_*CH_3_), 19.98 (NHCH_2_CH_2_C*H_2_*CH_3_), 31.52 (NHCH_2_C*H_2_*CH_2_CH_3_), 38.89 (NHC*H_2_*CH_2_CH_2_CH_3_), 40.24 (C*H_2_*CH_2_CH_3_), 68.15 (OC*H_2_*C=O), 114.04 (*C*H_arom._), 116.03* (C*H_arom._), 125.31 (*C*H_arom._), 131.22 (*C_q_*C=O), 149.12 (*C_q_*F or *C_q_*OCH_2_C=O), 153.30 (*C_q_*F or *C_q_*OCH_2_C=O), 166.69 (OCH_2_*C=*O), 197.84 (C_q_*C=*O).

*2-(4-Butyryl-2-fluorophenoxy)-N-(1-hydroxy-2-methylpropan-2-yl)acetamide* (**31**). Method B: Bromoacetyl bromide (0.76 mL, 8.75 mmol), 2-amino-2-methyl-1-propanol (780 mg, 8.75 mmol), triethylamine (1.23 mL, 8.75 mmol). Method C: 1-(3-Fluoro-4-hydroxyphenyl)butan-1-one (495 mg, 2.72 mmol), K_2_CO_3_ (563 mg, 4.08 mmol), KI (45.2 mg, 0.27 mmol), 2-bromo-*N*-(1-hydroxy-2-methylpropan-2-yl)acetamide (see method B). R_f_: 0.28 (cyclohexane/ethyl acetate: 1/2); yield 455 mg (1.46 mmol, 54 %); white solid; mp 84 °C (cyclohexane/ethyl acetate); ^1^H-NMR: δ 0.99 (t, 3H, ^3^*J* = 7.5 Hz, CH_2_CH_2_C*H_3_*), 1.35 (s, 6H, C_q_(C*H_3_*)_2_), 1.75 (sext, 2H, ^3^*J* = 7.4 Hz, CH_2_C*H_2_*CH_3_), 2.88 (t, 2H, ^3^*J* = 7.3 Hz, C*H_2_*CH_2_CH_3_), 3.65 (s, 2H, C_q_C*H_2_*OH), 4.52 (s, 2H, OC*H_2_*C=O), 6.66 (s, 1H, N*H*), 6.98 (dd, 1H, *J* = 8.1 Hz, C*H_arom._*), 7.73–7.76 (m, 2H, C*H_arom._*); ^13^C-NMR: δ 13.83 (CH_2_CH_2_*C*H_3_), 17.74 (CH_2_*C*H_2_CH_3_), 24.66 (C_q_(*C*H_3_)_2_), 40.26 (*C*H_2_CH_2_CH_3_), 56.50 (*C_q_*(CH_3_)_2_), 68.24 (O*C*H_2_C=O), 70.17 (C_q_*C*H_2_OH), 114.38 (*C*H_arom._), 116.30 (*C*H_arom._), 125.28 (*C*H_arom._), 132.21 (*C_q_*C=O), 148.87 (*C_q_*F or *C_q_*OCH_2_C=O), 153.34 (*C_q_*F or *C_q_*OCH_2_C=O), 167.42 (OCH_2_*C=*O), 197.85 (C_q_*C=*O).

*Methyl 2-(2-(4-butyryl-2-fluorophenoxy)acetamido)-3-methylpentanoate* (**32**). Method B: Bromoacetyl bromide (2.00 g, 9.92 mmol), isoleucine methylester HCl (1.80 mg, 9.92 mmol), triethylamine (2.79 mL, 19.8 mmol). Method C: 1-(3-Fluoro-4-hydroxyphenyl)butan-1-one (600 mg, 3.29 mmol), K_2_CO_3_ (683 mg, 4.94 mmol) KI (54.6 mg, 0.33 mmol), methyl-2-(2-bromoacetamido)-3-methyl-pentanoate (see method B)). R_f_: 0.65 (cyclohexane/ethyl acetate: 1/1); yield 616 mg (1.68 mmol, 51 %); white solid; mp 74 °C (cyclohexane/ethyl acetate); [α]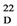 = 61.1 (CHCl_3_, c = 0.1); ^1^H-NMR: δ 0.90–0.94 (m, 6H, Ile-CH_2_C*H_3_* and Ile-CHC*H_3_*), 0.99 (t, 3H, ^3^*J* = 7.5 Hz, CH_2_CH_2_C*H_3_*), 1.12–1.23 (m, 2H, Ile-C*H_2_*CH_3_), 1.40–1.49 (m, 1H, Ile-C*H*C=O), 1.75 (sext, ^3^*J* = 7.3 Hz, CH_2_C*H_2_*CH_3_), 1.92–1.99 (m, 1H, Ile-C*H*CH_3_), 2.88 (t, 2H, ^3^*J* = 7.3 Hz, C*H_2_*CH_2_CH_3_), 3.74 (s, 3H, OC*H_3_*), 4.62 (s, 2H, OC*H_2_*C=O), 6.99 (dd, 1H, ^3^*J* = 8.5 Hz, C*H_arom._*), 7.08 (d, 1H, *J* = 8.5 Hz, N*H*), 7.72–7.77 (m, 2H, C*H_arom._*).

### Protein expression and purification

Falcipain-2 and falcipain-3 were recombinantly expressed in *E. coli* and refolded to active enzyme as previously described [13,24].

### Enzyme assays, in-vitro assays

IC_50_ values for inhibition of falcipain-2 and falcipain-3 were determined as described previously using the fluorogenic substrates Cbz-Phe-Arg-AMC and Cbz-Leu-Arg-AMC, respectively (AMC, 7-amino-4 methylcoumarin) [7, 13, 14]. As a positive control the well-known cysteine protease inhibitor E-64 [7, 25, 26] and as a negative control the solvent (DMSO) was used. Dose-dependent effects of compounds on parasite development (*P. falciparum* strain W2) were quantified by flow cytometry according to a previously published method [27]. First, a screening with inhibitor concentrations of 100, 10 and 1 mM was performed and the percentage activity of infected red blood cells (RBCs) relative to the negative control was determined. Compounds showing concentration dependent inhibition in these assays were selected for determination of IC_50_ values (Table 1). Chloroquine [20] and E-64 were used as positive controls, and the solvent DMSO was used as a negative control. For selected compounds assays with the *P. falciparum* strain 3D7 were performed using a fluorometric assay with Hoechst-33258 [28], or the microculture tetrazolium test measuring parasite lactate dehydrogenase activity [12, 29]. The parasites were cultivated *in vitro* as described previously [30]. Compounds were screened at concentrations between 1 nM and 100 μM. Synchronized ring stages were plated in 96-well plates at a parasitemia of 1%, in the presence of the compounds (dissolved in DMSO). Incubation of parasites with DMSO alone at a concentration of 0.5% v/v was used as a negative control. IC_50_ values were calculated by non-linear regression analyses using the programs GaphPad^®^ Prism 4 and GraFit^®^ [31, 32].

### In vitro cytotoxicity assay

The human kidney epithelium-cell line 293T was cultured in DMEM medium supplemented with 20% fetal bovine serum and 1% l-glutamine (200 mM) at 37 °C in a humidified atmosphere containing 5% CO_2_. For the experimental procedures, cells were detached from the flasks with a rubber policeman, washed twice with PBS (phosphate-buffer saline) containing 1 mL trypsin-EDTA (1 x), and suspended at 2 x 10^6^ cells mL^-1^ in complete medium. Inhibitor assays were carried out as described elsewhere except that the cell concentration used in this study was 2 x 10^4^ mL^-1^ well^-1^ [33].

## References

[1] WHO (2005). World Malaria Report.

[2] Talisuna A. O., Okello P. E., Erhart A., Coosemans M., D'Alessandro U. (2007). Intensity of malaria transmission and the spread of *Plasmodium falciparum* resistant malaria: a review of epidemiologic field evidence. Am. J. Trop. Med. Hyg..

[3] White N. J. (2004). Antimalarial drug resistance. J. Clin. Invest..

[4] Otto H.-H., Schirmeister T. (1997). Cysteine proteases and their inhibitors. Chem. Rev..

[5] Rosenthal P. J. (2004). Cysteine proteases of malaria parasites. Int. J. Parasitol..

[6] Rosenthal P. J., Sijwali P. S., Singh A., Shenai B. R. (2002). Cysteine proteases of malaria parasites: targets for chemotherapy. Curr. Pharm. Des..

[7] Dahl E. L., Rosenthal P. J. (2005). Biosynthesis, localization, and processing of falcipain cysteine proteases of *Plasmodium falciparum*. Mol. Biochem. Parasitol..

[8] Rupp I., Bosse R., Schirmeister T., Pradel G. (2008). Effect of protease inhibitors on exflagellation in *Plasmodium falciparum*. Mol. Biochem. Parasitol..

[9] Lavrado J., Paulo A., Gut J., Rosenthal P. J, Moreira R. (2008). Cryptolepine analogues containing basic aminoalkyl side-chains at C-11: synthesis, antiplasmodial activity, and cytotoxicity. Bioorg. Med. Chem. Lett..

[10] Vale N., Matos J., Gut J., Nogueira F., do Rosário V., Rosenthal P. J., Moreira R., Gomes P. (2008). Imidazolidin-4-one peptidomimetic derivatives of primaquine: synthesis and antimalarial activity. Bioorg. Med. Chem. Lett..

[11] Verissimo E., Berry N., Gibbons P., Cristiano M. L., Rosenthal P. J., Gut J., Ward S. A., O'Neill P. M. (2008). Design and synthesis of novel 2-pyridone peptidomimetic falcipain 2/3 inhibitors. Bioorg. Med. Chem. Lett..

[12] Ettari R., Nizi E., Di Francesco M. E., Dude M. A., Pradel G., Vicík R., Schirmeister T., Micale N., Grasso S., Zappalà M. (2008). Development of peptidomimetics with a vinyl sulfone warhead as irreversible falcipain-2 inhibitors. J. Med. Chem..

[13] Sijwali P. S., Shenai B. R.., Gut J., Singh A., Rosenthal P. J. (2001). Expression and characterization of the *Plasmodium falciparum* haemoglobinase falcipain-3. Biochem. J..

[14] Desai P. V., Patny A., Gut J., Rosenthal P. J., Tekwani B., Srivastava A., Avery M. (2006). Identification of novel parasitic cysteine protease inhibitors by use of virtual screening. 2. The available chemical directory. J. Med. Chem..

[15] Semenov A., Olson P. J., Rosenthal P. J. (1998). Antimalarial synergy of cysteine and aspartic protease inhibitors. Antimicrob. Agents Chemother..

[16] Kaeppler U., Schirmeister T. (2005). New non-peptidic inhibitors of papain derived from etacrynic acid. Med. Chem..

[17] Kaeppler U., Stiefl N., Schiller M., Vicik R., Breuning A., Schmitz W., Rupprecht D., Schmuck C., Baumann K., Ziebuhr J., Schirmeister T. J. (2005). A new lead for nonpeptidic active-site-directed inhibitors of the severe acute respiratory syndrome coronavirus main protease discovered by a combination of screening and docking methods. J. Med. Chem..

[18] Shenai B. R., Sijwali P. S., Singh A., Rosenthal P. J. (2000). Stage-specific antimalarial activity of cysteine protease inhibitors. J. Biol. Chem..

[19] Schulz F., Gelhaus C., Degel B., Vicik R., Heppner S., Breuning A., Leippe M., Rosenthal P. J., Schirmeister T. (2007). Screening of protease inhibitors as antiplasmodial agents. Part I: Aziridines and epoxides. ChemMedChem..

[20] Sijwali P. S., Koo J., Singh N., Rosenthal P. J. (2006). Gene disruptions demonstrate independent roles for the four falcipain cysteine proteases of *Plasmodium falciparum*. Mol. Biochem. Parasitol..

[21] Mikus J., Steverding D. (2000). A simple colorimetric method to screen drug cytotoxicity against *Leishmania* using the dye Alamar Blue. Parasitol. Int..

[22] Ponte-Sucre A., Faber J. H., Gulder T., Kajahn I., Pedersen S. E., Schultheis M., Bringmann G., Moll H. (2007). Activities of naphthylisoquinoline alkaloids and synthetic analogs against *Leishmania major*. Antimicrob. Agents Chemother..

[23] Gelhaus C., Vicik R., Hilgenfeld R., Schmidt C. L., Leippe M., Schirmeister T. (2004). Synthesis and antiplasmodial activity of a cysteine protease-inhibiting biotinylated aziridine-2,3-dicarboxylate. Biol. Chem..

[24] Pandey K. C., Wang S. X., Sijwali P. S., Lau A. L., McKerrow J. H., Rosenthal P. J. (2005). The *Plasmodium falciparum* cysteine protease falcipain-2 captures its substrate, hemoglobin, via a unique motif. Proc. Natl. Acad. Sci. USA.

[25] Ramjee M. K., Flinn N. S., Pemberton T. P., Quibell M., Wang Y., Watts J. P. (2006). Substrate mapping and inhibitor profiling of falcipain-2, falcipain-3 and berghepain-2: implications for peptidase anti-malarial drug discovery. Biochem. J..

[26] Sijwali P. S., Rosenthal P. J. (2004). Gene disruption confirms a critical role for the cysteine protease falcipain-2 in hemoglobin hydrolysis by *Plasmodium falciparum*. Proc. Natl. Acad. Sci. USA.

[27] Musonda C. C., Gut J., Rosenthal P. J., Yardley V., Carvalho de Souza R. C., Chibale K. (2006). Application of multicomponent reactions to antimalarial drug discovery. Part 2: New antiplasmodial and antitrypanosomal 4-aminoquinoline gamma- and delta-lactams via a 'catch and release' protocol. Bioorg. Med. Chem..

[28] Smeijsters L. J., Zijlstra N. M., Franssen F. F., Overdulve J. P. (1996). Simple, fast, and accurate fluorometric method to determine drug susceptibility of *Plasmodium falciparum* in 24-well suspension culture. Antimicrob. Agents Chemother..

[29] Makler M. T., Hinrichs D. J. (1993). Measurement of the lactate dehydrogenase activity of *Plasmodium falciparum* as an assessment of parasitemia. Am. J. Trop. Med. Hyg..

[30] Ifediba T., Vanderberg J. P. (1981). Complete *in vitro* maturation of *Plasmodium falciparum* gametocytes. Nature.

[31] (2003). GraphPad^®^ Prism 4.

[32] (2006). GraFit^®^ 5.0.13.

[33] Ahmed S. A., Gogal R. M., Walsh J. E. (1994). A new rapid and simple non-radioactive assay to monitor and determine the proliferation of lymphocytes: an alternative to [3H]thymidine incorporation assay. J. Immunol. Methods.

